# Supplementary material for: Incidence of Diabetic Retinopathy in Individuals with Type 2 Diabetes: A Study Using Real-World Data
Source: J Clin Med. 2024 Nov 23;13(23):7083. doi: 10.3390/jcm13237083 (PMC11642251; doi:10.3390/jcm13237083)
Supplement: Supplementary file 1 [file jcm-13-07083-s001.zip › jcm-3287946-supplementary.pdf]

Incidence of diabetic retinopathy in individuals with type 2 diabetes: A real-world data study

Carlos Hernández-Teixidó; Joan Barrot de la Puente; Sònia Miravet Jiménez; Berta Fernández Camins ; Didac Mauricio ; Pere Romero Aroca; Bogdan Vlacho ; Josep Franch-Nadal

Title	page
Figure S1: Study flowchart	2
Table S1. Clinical characteristics of the subjects at inclusion according to subsequent development of different forms of DR	3
Table S2. Incidence of MILD NPDR for risk factors	4
Table S3. Incidence of MODERATE NPDR for risk factors	5
Table S4. Incidence of SEVERE NPDR for risk factors	6
Table S5. Incidence of PDR for risk factors	7
Table S6. Incidence of DME for risk factors	8
Table S7: HRs for incidence DR adjusted by different clinically important variables	9

Figure S1: Study flowchart

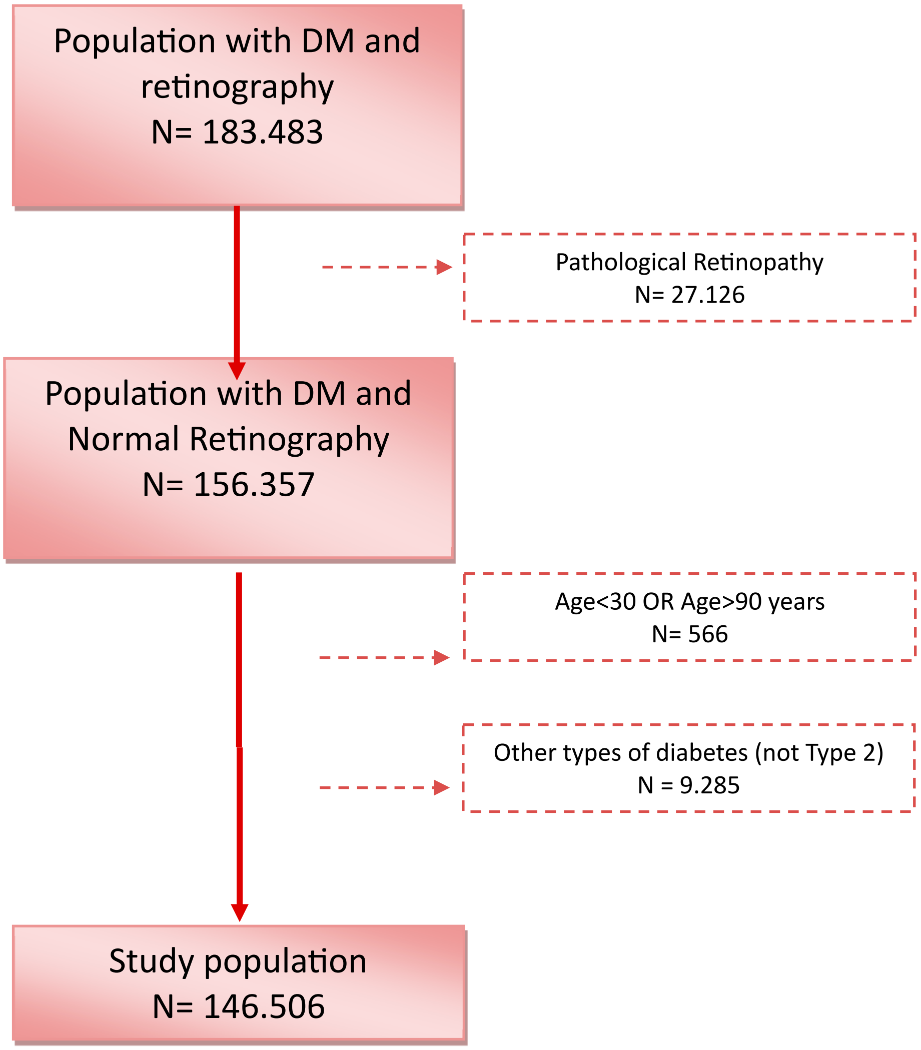

Table S1. Clinical characteristics of the subjects at inclusion according to subsequent development of different forms of DR

Variables	NDR [N= 105446]	Mild NPDR [N= 4946]	Moderate NPDR [N= 1482]	Severe NPDR [N= 148]	PDR [N= 166]	MDE [N= 139]	p
Age (years), mean (SD)	64.1 (10.5)	65.1 (11.0)	66.3 (11.3)	63.6 (13.0)	67.9 (11.3)	67.0 (11.3)	<0.001
Non smoker	54914 (53.3%)	2543 (52.5%)	755 (52.1%)	66 (45.2%)	79 (50.3%	83 (61.0%)	0.101
Current smoker, N (%)	17895 (17.4%)	812 (16.58%)	243 (16.8%)	34 (23.3%)	29 (18.5%)	22 (16.2%)	
Ex smoker, N (%)	30165 (29.3%)	1487 (30.7%)	450 (31.1%)	46 (31.5%)	49 (31.2%)	31 (22.8%)	
**Clinical variables, mean (SD)**							
Diabetes duration, mean (SD)	5.3 (4.8)	7.5 (5.7)	7.7 (5.58)	7.6 (5.1)	10.1 (7.0)	6.1 (4.9)	<0.001
BMI (kg/m²)	30.6 (5.0)	30.4 (5.1)	30.3 (5.0)	29.7 (5.4)	30.2 (4.7)	30.3 (4.7)	0.002
SBP (mmHg)	134 (14.1)	136 (15.5)	136 (15.9)	136 (13.7)	137 (16.3)	137 (14.7)	<0.001
DBP (mmHg)	77.0 (9.3)	76.9 (9.8)	76.2 (10.3)	77.2 (11.4)	74.6 (10.6)	76.6 (10.5)	0.001
**Laboratory variables, mean (SD)**							
HbA1c (%)	7.1 (1.3)	7.8 (1.7)	8.1 (1.8)	8.8 (2.3)	7.98 (1.7)	7.7 (1.9)	0.000
Total Cholesterol (mg/dL)	193 (39.6)	190 (41.6)	193 (45.2)	199 (51.3)	184 (36.1)	193 (43.5)	<0.001
HDL Cholesterol (mg/dL)	48.7 (12.7)	48.2 (12.7)	48.3 (13.6)	46.0 (9.8)	48.8 (14.0)	49.6 (12.6)	0.029
LDL Cholesterol (mg/dL)	114 (33.6)	111 (33.9)	112 (36.5)	113 (38.5)	106 (31.9)	113 (35.6)	<0.001
Triglycerides (mg/dL)	165 (117)	166 (117)	171 (118)	210 (194)	158 (120)	170 (145)	0.001
GFR (CKD-EPI; ml/min/1.73m²)	58.7 (4.7)	58.1 (5.9)	58.0 (6.1)	57.0 (7.3)	55.8 (9.8)	57.9 (6.6)	<0.001
Album-to-creatinine ratio	24.3 (99.1)	44.3 (158)	48.9 (181)	29.9 (54.3)	81.2 (216)	44.8 (206)	<0.001
**Comorbidities, N (%)**							
Dyslipidemia	66060 (62.6%)	3110 (62.9%	925 (62.4%)	93 (62.8%)	106 (63.69%)	98 (70.5%)	0.564
Hypertension	68964 (65.4%)	3267 (66.1%)	996 (67.2%)	90 (60.8%)	127 (76.5%)	95 (68.3%)	0.017
Chronic Kidney disease (FG<60 or CAC>30)	18484 (19.7%)	1164 (26.9%)	360 (27.5%)	39 (32.8%)	59 (41.8%)	37 (29.1%)	<0.001
Coronary heart disease	8514 (8.0%)	512 (10.4%)	159 (10.7%)	15 (10.1%)	24 (14.5%)	6 (4.3%)	<0.001
Stroke	5227 (4.9%)	301 (6.0%)	87 (5.8%)	10 (6.7%)	17 (10.2%)	11 (7.9%)	<0.001
Peripheral artery disease	3204 (3.0%)	250 (5.0%)	76 (5.1%)	7 (4.7%)	13 (7.8%)	5 (3.6%)	.
Heart failure	2856 (2.7%)	182 (3.6%)	69 (4.6%)	6 (4.0%)	11 (6.6%)	4 (2.8%)	.
**Concomitant treatment, N(%)**							
Antihypertensive drugs	75427 (71.5%)	3640 (73.6%)	1126 (76.0%)	92 (62.2%)	135 (81.3%)	101 (72.7%)	<0.001
Antidiabetics drugs:							.
- No drugs	18266 (17.3%)	424 (8.5%)	126 (8.5%)	9 (6.0%)	7 (4.2%)	18 (12.9%)	
- NIAD combo	74248 (70.4%)	3059 (61.8%)	899 (60.7%)	88 (59.5%)	79 (47.6%)	91 (65.5%)	
- NIAD + INS	11354 (10.8%)	1285 (26.0%)	403 (27.2%)	42 (28.4%)	70 (42.2%)	24 (17.3%)	
- INS mono	1578 (1.5%)	178 (3.6%)	54 (3.6%)	9 (6.0%)	10 (6.0%)	6 (4.3%)	

SD, standard deviation; BMI, body mass index; SBP, systolic blood pressure; DBP, diastolic blood pressure; HbA1c, glycosylated hemoglobin; SD, standard deviation; Glomerular filtration rate, GFR; Albumin to Creatinine ratio, CAC NIAD: non-insulin antidiabetic drugs; INS: insulins

Table S2 . Incidence of <u>MILD NPDR</u> for risk factors

Group of subjects	Patients (number)	Sum follow-up	Follow-up (mean)	Follow-up (median)	Events (number)	Incidence rate per 1000 person-years	Cumulative incidence	Hazard ratio (95%CI)
**Female**	62586	431650.9	6.90	7.14	2036	4.72	3.25	Ref.
**Male**	83920	561379.0	6.69	6.92	2910	5.18	3.47	1.09 (1.03; .15)
**Smoker**								
Non smoker	75770	521033.8	6.88	7.12	2543	4.88	3.36	Ref.
Ex smoker	43020	281933.0	6.55	6.80	1487	5.27	3.46	1.06 (1.00; 1.13)
Current smoker	24335	163987.7	6.74	6.91	812	4.95	3.34	1.00 (0.93; 1.09)
**Diabetes duration**								
0 – 5 years	79526	542781.1	6.83	6.97	1902	3.50	2.39	Ref.
6 – 10 years	45445	312439.5	6.88	7.24	1809	5.79	3.98	1.68 (1.58:1.79)
>10 years	21535	137809.3	6.40	6.63	1235	8.96	5.73	2.52 (2.35; 2.71)
**HbA1c (%)**								
<7%	70255	463530.3	6.60	6.76	1624	3.50	2.31	Ref.
7 – 8%	33825	225396.6	6.66	6.93	1179	5.23	3.49	1.51 (1.40; 1.63)
8.1 – 10%	18316	122112.8	6.67	7.01	980	8.03	5.35	2.33 (2.15: 2.52)
>10%	6654	43101.0	6.48	6.76	538	12.48	8.09	3.57 (3.24; 3.93)
**Obesity**								
BMI ≤ 30kg/m^2	64253	422842.2	6.58	6.80	2158	5.10	3.36	Ref.
BMI > 30kg/m^2	60669	405451.3	6.68	6.86	1949	4.81	3.21	0.94 (0.89; 1.00)
**Comorbidities**								
Non hypertension	48652	337095.3	6.93	7.18	1679	4.98	3.45	Ref.
Hypertension	97854	655934.6	6.70	6.93	3267	4.98	3.34	0.99 (0.93; 1.05)
Non macrovascular disease	122397	841362.6	6.87	7.11	4032	4.79	3.29	Ref.
Macrovascular disease	24109	151667.3	6.29	6.52	914	6.03	3.79	1.22 (1.14; 1.31)
Non chronic kidney disease	100412	675698.0	6.73	6.92	3170	4.69	3.16	Ref.
Chronic kidney disease	30239	187431.3	6.20	6.42	1164	6.21	3.85	1.30 (1.21; 1.39)

HbA1c, glycosylated hemoglobin; BMI, body mass index; SD, standard deviation.

Table S3. Incidence of MODERATE NPDR for risk factors

Group of subjects	Patients (number)	Sum follow-up	Follow-up (mean)	Follow-up (median)	Events (number)	Incidence rate per 1000 person-years	Cumulative incidence	Hazard ratio (95%CI)
**Female**	62586	438143.9	7.00	7.20	605	1.38	0.97	Ref.
**Male**	83920	570423.5	6.80	6.99	877	1.54	1.05	1.10 (0.99; 1.22)
**Smoker**								
Non smoker	75770	529206.3	6.98	7.19	755	1.43	1.00	Ref.
Ex smoker	43020	286413.8	6.66	6.88	450	1.57	1.05	1.09 (0.97; 1.22)
Current smoker	24335	166525.9	6.84	6.98	243	1.46	1.00	1.01 (0.88; 1.17)
**Diabetes duration**								
0 – 5 years	79526	548819.2	6.90	7.04	545	0.99	0.69	Ref.
6 – 10 years	45445	318248.2	7.00	7.36	560	1.76	1.23	1.80 (1.60; 2.02)
>10 years	21535	141499.9	6.57	6.78	377	2.66	1.75	2.66 (2.33; 3.03)
**HbA1c (%)**								
<7%	70255	468728.4	6.67	6.81	407	0.87	0.58	Ref.
7 – 8%	33825	229047.6	6.77	7.01	336	1.47	0.99	1.71 (1.48; 1.97)
8.1 – 10%	18316	125021.4	6.83	7.11	326	2.61	1.78	3.05 (2.64; 3.53)
>10%	6654	44485.4	6.69	5.89	230	5.17	3.46	5.98 (5.09; 7.03)
**Obesity**								
BMI ≤ 30kg/m²	64253	429354.6	6.68	6.87	657	1.53	1.02	Ref.
BMI > 30kg/m²	60669	411417.0	6.78	6.91	582	1.41	0.96	0.92 (0.83; 1.03)
**Comorbidities**								
Non hypertension	48652	342508.0	7.04	7.26	486	1.42	1.00	Ref.
Hypertension	97854	666059.4	6.71	7.01	996	1.50	1.02	1.04 (0.94; 1.16)
Non macrovascular disease	122397	854191.4	6.98	7.18	1206	1.41	0.99	Ref.
Macrovascular disease	24109	154376.0	6.40	6.62	276	1.79	1.14	1.24 (1.09; 1.41)
Non chronic kidney disease	100412	685373.4	6.83	6.98	948	1.38	0.94	Ref.
Chronic kidney disease	30239	190853.8	6.31	6.50	360	1.89	1.19	1.34 (1.19; 1.51)

HbA1c, glycosylated hemoglobin; BMI, body mass index; SD, standard deviation.

Table S4. Incidence of SEVERE NPDR for risk factors

Group of subjects	Patients (number)	Sum follow-up	Follow-up (mean)	Follow-up (median)	Events (number)	Incidence rate per 1000 person–years	Cumulative incidence	Hazard ratio (95%CI)
**Female**	62586	40595.1	7.04	7.24	55	0.12	0.09	Ref.
**Male**	83920	573733.6	6.84	7.04	93	0.16	0.11	1.30 (0.93; 1.81)
**Smoker**								
Non smoker	75770	532229.5	7.02	7.21	66	0.12	0.09	Ref.
Ex smoker	43020	288160.4	6.70	6.91	46	0.16	0.11	1.28 (0.88; 1.87)
Current smoker	24335	167389.2	6.88	7.03	34	0.20	0.14	1.64 (1.08; 2.47)
**Diabetes duration**								
0 – 5 years	79526	550872.6	6.93	7.06	47	0.09	0.06	Ref.
6 – 10 years	45445	320517.5	7.05	7.43	68	0.21	0.15	2.50 (1.72; 3.62)
>10 years	21535	142938.5	6.64	6.84	33	0.23	0.15	2.70 (1.73; 4.21)
**HbA1c (%)**								
<7%	70255	470370.4	6.70	6.83	34	0.07	0.05	Ref.
7 – 8%	33825	230390.4	6.81	7.05	18	0.08	0.05	1.08 (0.61; 1.91)
8.1 – 10%	18316	126199.6	6.89	7.15	33	0.26	0.18	3.64 (2.25; 5.87)
>10%	6654	45286.9	6.81	6.97	34	0.75	0.51	10.4 (6.47; 16.7)
**Obesity**								
BMI ≤ 30kg/m²	64253	431804.1	6.72	6.90	66	0.15	0.10	Ref.
BMI > 30kg/m²	60669	413695.2	6.82	6.93	50	0.12	0.08	0.79 (0.55; 1.14)
**Comorbidities**								
Non hypertension	48652	344351.45	7.08	7.28	58	0.17	0.12	Ref.
Hypertension	97854	669977.3	6.85	7.05	90	0.13	0.09	0.80 (0.57; 1.11)
Non macrovascular disease	122397	858934.1	7.02	7.20	123	0.14	0.10	Ref.
Macrovascular disease	24109	155394.6	6.45	6.65	25	0.16	0.10	1.12 (0.73; 1.72)
Non chronic kidney disease	100412	689048.3	6.86	7.03	80	0.12	0.08	Ref.
Chronic kidney disease	30239	192150.8	6.35	6.55	39	0.20	0.13	1.75 (1.19: 2.56)

HbA1c, glycosylated hemoglobin; BMI, body mass index; SD, standard deviation.

Table S5. Incidence of PDR for risk factors

Group of subjects	Patients (number)	Sum follow-up	Follow-up (mean)	Follow-up (median)	Events (number)	Incidence rate per 1000 person–years	Cumulative incidence	Hazard ratio (95%CI)
**Female**	62586	440486.2	7.04	7.23	73	0.17	0.12	Ref.
**Male**	83920	573683.7	6.84	7.04	93	0.16	0.11	0.97 (0.72; 1.32)
**Smoker**								
Non smoker	75770	532125.1	7.02	7.21	79	0.15	0.10	Ref.
Ex smoker	43020	288129.37	6.70	6.91	49	0.17	0.11	1.14 (0.80; 1.62)
Current smoker	24335	167387.5	6.88	7.03	29	0.17	0.12	1.16 (0.76; 1.77)
**Diabetes duration**								
0 – 5 years	79526	550877.2	6.93	7.06	48	0.09	0.06	Ref.
6 – 10 years	45445	550877.2	7.05	7.43	52	0.16	0.11	1.88 (1.27; 2.79)
>10 years	21535	142766.0	6.63	6.84	66	0.46	0.31	5.28 (3.64; 7.65)
**HbA1c (%)**								
<7%	70255	470323.6	6.69	6.83	43	0.09	0.06	Ref.
7 – 8%	33825	230276.2	6.81	7.05	43	0.19	0.13	2.06 (1.35; 3.14)
8.1 – 10%	18316	126165.8	6.89	7.15	36	0.29	0.20	3.15 (2.02; 4.91)
>10%	6654	45293.2	6.81	6.97	20	0.44	0.30	4.85 (2.86; 8.25)
**Obesity**								
BMI ≤ 30kg/m^2	64253	431745.5	6.72	6.90	67	0.16	0.10	Ref.
BMI > 30kg/m^2	60669	413615.9	6.82	6.93	63	0.15	0.10	0.98 (0.70; 1.39)
**Comorbidities**								
Non hypertension	48652	344409.7	7.08	7.28	39	0.11	0.08	Ref.
Hypertension	97854	669760.2	6.84	7.05	127	0.19	0.13	1.66 (1.16; 2.38)
Non macrovascular disease	122397	858872.5	7.02	7.20	121	0.14	0.10	Ref.
Macrovascular disease	24109	155297.4	6.44	6.65	45	0.29	0.19	2.02 (1.44; 2.86)
Non chronic kidney disease	100412	688993.5	6.86	7.03	82	0.12	0.08	Ref.
Chronic kidney disease	30239	192004.3	6.35	6.55	59	0.31	0.20	2.55 (1.82; 3.56)

HbA1c, glycosylated hemoglobin; BMI, body mass index; SD, standard deviation.

Table S6. Incidence of DME for risk factors

Group of subjects	Patients (number)	Sum follow-up	Follow-up (mean)	Follow-up (median)	Events (number)	Incidence rate per 1000 person–years	Cumulative incidence	Hazard ratio (95%CI)
**Female**	62586	440580.4	7.04	7.24	52	0.12	0.08	Ref.
**Male**	83920	573803.4	6.84	7.04	87	0.15	0.10	1.28 (0.91; 1.80)
**Smoker**								
Non smoker	75770	532162.2	7.02	7.21	83	0.16	0.11	Ref.
Ex smoker	43020	288222.5	6.70	6.91	31	0.11	0.07	0.69 (0.45; 1.04)
Current smoker	24335	167444.5	6.88	7.03	22	0.13	0.09	0.84 (0.52; 1.34)
**Diabetes duration**								
0 – 5 years	79526	550826.7	6.93	7.06	66	0.12	0.08	Ref.
6 – 10 years	45445	320563.7	7.05	7.43	47	0.15	0.10	1.24 (0.85; 1.80)
>10 years	21535	142993.3	6.64	6.84	26	0.18	0.12	1.51 (0.96; 2.38)
**HbA1c (%)**								
<7%	70255	470319.6	6.69	6.83	55	0.12	0.08	Ref.
7 – 8%	33825	230307.5	6.81	7.05	35	0.15	0.10	1.31 (0.86; 2.00)
8.1 – 10%	18316	126259.9	5.89	7.15	22	0.17	0.12	1.50 (0.92; 2.47)
>10%	6654	45334.1	6.81	6.98	14	0.31	0.21	2.65 (1.48; 4.77)
**Obesity**								
BMI ≤ 30kg/m^2	64253	431806.8	6.72	6.90	66	0.15	0.10	Ref.
BMI > 30kg/m^2	60669	413688.9	6.82	6.93	52	0.13	0.09	0.82 (0.57; 1.18)
**Comorbidities**								
Non hypertension	48652	344407.9	7.08	7.28	44	0.13	0.09	Ref.
Hypertension	97854	669975.8	6.85	7.05	95	0.14	0.10	1.11 (0.77; 1.58)
Non macrovascular disease	122397	858952.2	7.02	7.20	119	0.14	0.10	Ref.
Macrovascular disease	24109	155431.5	6.45	6.65	20	0.13	0.08	0.92 (0.57; 1.48)
Non chronic kidney disease	100412	688996.4	6.86	7.03	90	0.13	0.09	Ref.
Chronic kidney disease	30239	192162.8	6.35	6.55	37	0.19	0.12	1.46 (1.00; 2.15)

HbA1c, glycosylated hemoglobin; BMI, body mass index; SD, standard deviation.

Table 7: HRs for incidence of DR adjusted by different clinically important variables

	Model 1 (HR 95%CI)	Model 2 (HR 95%CI)	Model 3 (HR 95%CI)
**Age (years)**	1.004 (1.002; 1.006)	1.004 (1.002; 1.007)	1.003 (1.000; 1.006)
**Sex (male)**	1.108 (1.056; 1.163)	1.107 (1.051; 1.166)	1.082 (1.017; 1.151)
**Duration T2DM (years)**		1.052 (1.048; 1.056)	1.050 (1.046; 1.054)
**HbA1c (%)**		1.311 (1.294; 1.329)	1.306 (1.287; 1.325)
**Ex-smoker (ref: Non-smoker)**			1.007 (0.942; 1.075)
**Smoker (ref: Non-smoker)**			0.970 (0.893; 1.055)
**Body mass index (kg/m^2)**			0.997 (0.992; 1.003)
**Hypertension (ref: No)**			1.004 (0.943; 1.069)
**Dyslipidemia (ref: No)**			0.978 (0.924; 1.036)
**Macrovascular disease (ref: No)**			1.143 (1.064; 1.228)

**Macrovascular disease: peripheral arteriopathy or AVC or ischemic cardiopathy*

Model 1: adjusted for age and sex; Model 2: adjusted for, age, sex, diabetes duration, HbA1c; Model 3: adjusted for age, sex, diabetes duration, HbA1c, smoking status, BMI, hypertension, dyslipidemia, and macrovascular disease.
